# Quality of life during the COVID-19 pandemic in Austria

**DOI:** 10.3389/fpsyg.2022.934253

**Published:** 2022-08-01

**Authors:** Rachel Dale, Sanja Budimir, Thomas Probst, Elke Humer, Christoph Pieh

**Affiliations:** ^1^Department for Psychosomatic Medicine and Psychotherapy, University for Continuing Education Krems Krems, Krems, Austria; ^2^Department of Work, Organization and Society, Ghent University, Ghent, Belgium

**Keywords:** quality of life, COVID-19, Austria, lockdown, urbanization, gender, age

## Abstract

The COVID-19 pandemic has had unprecedented effects on our daily lives. This study aimed to assess the quality of life (QoL) (WHOQOL-Bref physical, social, and environmental domains) at two time points during the COVID-19 pandemic with lockdown restrictions according to gender, age, and urbanization level. Qualtrics^®^ recruited representative Austrian population samples in April 2020 (t1; *N* = 1,005) and December 2020/January 2021 (t2; *N* = 1,505). ANOVAs and the Bonferroni-corrected *post-hoc* tests were conducted to investigate differences between April and December 2020 and to compare with pre-pandemic data. Although the quality of life (physical, social, and environmental domains) changed from pre-pandemic (mean scores 80, 77, and 81, respectively) to April 2020 (mean scores 72, 65, and 75, all *p*-values < 0.001), there were no significant changes between April and December (mean scores 75, 65, and 75). Living location (urban vs. rural), gender, and age showed an effect on the quality of life. All domains of quality of life have decreased since the onset of the pandemic, and this decline has been maintained over the course of the first year of the pandemic. Creative measures should be implemented to assist people in improving one or more areas of quality of life, within the lockdown restrictions to improve the overall wellbeing of the population.

## Introduction

Quality of life (QoL) is defined by the World Health Organization (WHO) as “individuals' perceptions of their position in life in the context of the culture and value systems in which they live and in relation to their goals, expectations, standards, and concerns” [(WHO, 1996), p. 3]. Due to the multi-dimensional nature of “quality of life,” it is usually measured in a multi-faceted manner. For example, the WHOQOL-BREF (WHO, 1996) measures the quality of life on four domains, rather than as a single, overall score:

1. Physical—Pain and Discomfort, Energy and Fatigue, Sleep and Rest, Medication Dependence, Mobility, Activities of Daily Living, and Working Capacity.
2. Psychological—Bodily Image and Appearance, Negative Feelings, Positive Feelings, Self-Esteem, Spirituality/Religion/Personal Beliefs, and Thinking/Learning/Memory/Concentration.
3. Social—Personal Relationships, Sexual Activity, and Social Support.
4. Environment—Financial Resources, Information and Skills Opportunities, Recreation and Leisure, Home Environment, Access and Quality of Health and Social Care, Freedom, Physical Safety and Security, Physical Environment (e.g., Pollution, Noise, and Climate), and Transport.

Each of these domains plays a role in a person's overall satisfaction with their lives and surroundings and as such, quality of life is an integral measure of general life satisfaction (Moons et al., 2006). Thus, this measure can be used to assess the impact the COVID-19 pandemic has had on the overall life quality of the general population. At the societal level, Europe has enjoyed a period of stability regarding safety, opportunities, and resources (Maier, 1981), which the COVID-19 pandemic has thrown into question. For individuals, COVID-19 has resulted in a reduction in many activities of daily life; physically, socially, and occupationally, which are major components of quality of life. Furthermore, extensive research has shown an increase in mental illness symptoms during COVID-19 (Vindegaard and Benros, 2020; de Sousa Júnior et al., 2021; Robinson et al., 2021). Therefore, a reduction in quality of life during the pandemic, and particularly during lockdown restrictions, would be expected, as has been observed in previous pandemics (Kwek et al., 2006; Matua and van der Wal, 2015).

Indeed, the few studies comparing the quality of life during COVID-19 with pre-COVID-19 data have demonstrated a decrease in quality of life since the onset of the pandemic in populations in Italy (Epifanio et al., 2021), Portugal (Ferreira et al., 2021), and South Korea (Park et al., 2021). Although the pre-pandemic comparisons were based on self-reports from memory in the latter and much older data in the study from Italy, the findings nonetheless suggest an expected impact of COVID-19 on QoL in disparate cultures. More research in other countries would build a fuller picture of the quality of life in general populations during the pandemic.

This study aimed to investigate the extent to which quality of life has been affected by the pandemic in Austria by comparing pre-pandemic data with two time points during the pandemic; early on and at the end of 2020. Furthermore, we explored which aspects of QoL, in which groups of the population, were most affected. This is important to mitigate as many negative effects of the pandemic as possible, to identify aspects of life that are important to people that could be improved within the boundaries of the necessary governmental restrictions, and to direct appropriate resources to those most burdened. In other aspects of mental health, women and young people have been more burdened by the pandemic (Salari et al., 2020; Vindegaard and Benros, 2020), including the Austrian population (Pieh et al., 2020; Dale et al., 2021; Humer et al., 2021) and in quality of life (Horesh et al., 2020). As such, we focused on age and gender in our analyses of changes over time. Furthermore, urban vs. countryside environments present characteristics that contribute to health and wellbeing during a pandemic, such as access to facilities, green space, and urban density (Mouratidis and Yiannakou, 2022). Therefore, it is conceivable that they have experienced disparate impacts on quality of life due to the pandemic. Indeed those in urban environments showed a decline in life satisfaction after the onset of the pandemic in Greece (Mouratidis and Yiannakou, 2022), however, there was no rural comparator in this study. To the best of our knowledge, there is no data on the quality of life in urban areas compared with rural inhabitants during the pandemic. Thus, we also explored whether urbanization affected the quality of life.

To investigate the above aims, we divided the study into two research questions: (1) how has QoL changed over time from pre-pandemic data and over the course of the pandemic, and is this affected by age and gender? (2) Is QoL during COVID-19 affected by the urbanization levels of the living environment?

To investigate question one, we asked a representative sample of the Austrian population to report on their quality of life during two lockdowns in April 2020 (hereafter referred to as April) and December 2020/January 2021 (hereafter referred to as Dec/Jan). These results were compared with pre-pandemic data from a large-scale representative survey collected by Austria's Federal Statistical Office in 2018–2019 (Statistik Austria, 2020). Results from the psychological domain of the WHOQOL-BREF have been published elsewhere as a measure of mental health (Pieh et al., 2020, 2021a; Dale et al., 2021). Therefore, this research question will predominantly focus on the other aspects of QoL. We predicted an overall decline in quality of life from pre-pandemic to pandemic times. However, few differences have been observed in other mental health measures between April and Dec/Jan (Dale et al., 2021) and therefore, we expected no further decline in QoL between the two pandemic time points. We predicted women and younger age groups would have poorer QoL during pandemic time points than men and older age groups, respectively.

For question two, only the Dec/Jan sample was asked about the level of urbanization, and therefore we considered them in isolation. Results from the psychological domain regarding urbanization have not been previously published, and therefore all four domains were included in these analyses. Given the lack of data on urbanization and QoL during the pandemic, we did not make any specific predictions.

## Materials and methods

### Study design and sample

Two sets of respondents were recruited and surveyed online by Qualtrics^®^ (Qualtrics, 2019) between the 10 and 30 April 2020 (*N* = 1,005) and between the 23 December 2020 and 4 January 2021 (*N* = 1,505). During both time points, COVID-19 cases were high in Austria [first and second waves, respectively (John Hopkins University, 2022)] and lockdowns were in place, meaning that people could only leave their homes for essential reasons; averting immediate danger to life, bodily harm, or property; professional activity (if home-office is not possible); errands to cover necessary basic needs; care and assistance for people in need of support; and exercising outdoors. A distance of at least 1 m to other people had to be ensured. In Austria, people were allowed to meet in groups of up to 10 people on the 24 and 25 December 2020 but no group gatherings were allowed at other times, including on the 31 December 2020. Demographic characteristics of the study samples can be found in the Supplementary Table S1. Participants had to be over 18 years old, have an adequate level of German, and be resident in Austria. Representative samples according to gender, age, region, and education level were calculated by Qualtrics^®^ and aimed for. Due to the limited time period of the second survey, not all quotas were met (as shown in Supplementary Table S2).

### Measures and variables

Quality of life was measured with the WHOQOL-BREF (WHO, 1996) at all time points. The 26-item self-rating questionnaire asks about the past 2 weeks and is divided into four domains: physical, psychological, social, and environment. This measure is validated and has good to excellent psychometric properties of reliability (Skevington et al., 2004). Results in the psychological domain, i.e., the effect of time point, gender, and age have been published previously (Pieh et al., 2020, 2021a; Dale et al., 2021). Therefore, the psychological domain is only included in the analyses on the effect of urbanization in the current article. Cronbach's alphas for the April sample were: physical α = 0.79, social α = 0.72, and environment α = 0.79, and for the Dec/Jan sample were: physical α = 0.84, social α = 0.76, environment α = 0.80, and psychological α = 0.85.

Gender was coded as male/female/non-binary. No participants identified as non-binary. Age was coded into six categories: 18–24, 25–34, 35–44, 45–54, 55–64, and 65+ years. Urbanization was only included in the Dec/Jan survey and was divided into two categories: village/small town (<25,000 inhabitants) and city (>25,000 inhabitants). The category was self-reported by participants. Population density was differently considered in the pre-pandemic data (Statistik Austria, 2020), which had three categories: low (<5,000 people), middle (5,000–50,000), and high (>50,000), and therefore the scores from the two samples were not directly compared statistically.

### Statistical analyses

Data were analyzed using SPSS version 27 (IBM, 2020) and graphics were produced in R version 4.0.3 (R Core Team, 2015) using the ggplot2 package (Wickham, 2016). ANOVAs were conducted to investigate differences between the three time points (pre-pandemic, April, and Dec/Jan) on each QoL domain, and the Bonferroni-corrected *post-hoc* tests were performed to investigate significant main effects. The *p*-values <0.05 were considered statistically significant (2-sided tests). The pre-pandemic sample was from a large-scale representative survey collected by Austria's Federal Statistical Office in 2018–2019 (Statistik Austria, 2020) and consisted of 15,461 participants but was weighted to produce a statistical sample of 7.4 million, representing Austria's over 15s population at the time of data collection (Statistik Austria, 2020).

## Results

A total of *N* = 1,005 respondents participated at t1 (April 2020, 52.7% women) and *N* = 1,505 at t2 (Dec 2020/Jan 2021, 49.2% women). Details of the socio-demographic characteristics of the study samples can be found in the Supplementary Table S1.

### Research question 1

#### Overall

In the full sample, the pre-pandemic quality of life scores were between 77 and 81 (from a maximum of 100). This is in contrast to the scores during the pandemic, which were 65–75 in April and 65–75 in Dec/Jan. Full descriptive results are shown in Table 1. There was a significant effect of time period on all measures of quality of life [Physical: *F*(2, 7.420, 293) = 123.36, *p* < 0.001, Social F (2, 7,420, 382) = 498.21, *p* < 0.001, Environmental: *F*(2, 7.420, 293) = 253.09, *p* < 0.001]. Social QoL and Environmental QoL were significantly worse in both lockdowns compared with the pre-pandemic data (all *p* < 0.001), but there is no significant difference between April and Dec/Jan (all *p* = 1.0). Physical QoL was significantly worse in both lockdowns compared with the pre-pandemic data (*p* < 0.001). Interestingly, it was significantly better in Dec/Jan than in April (*p* = 0.001).

**Table 1:** Mean (SD) quality of life scores at each time point for the whole sample and according to gender.

	**Pre-pandemic**			**April lockdown 2020**			**Dec20/Jan21 lockdown**			**Statistic**
	**Male**	**Female**	**Total**	**Male**	**Female**	**Total**	**Male**	**Female**	**Total**	
Physical	81.37 (17.6)	78.3 (19.3)	79.8 (18.5)	74.4 (17.6)	70.36 (16.8)	72.31 (17.3)	77.07 (16.8)	73.17 (19.0)	75.09 (18.1)	Gender: *F*(1) = 50.56, *p* < 0.001
Social	77.15 (19.4)	77.32 (19.7)	77.23 (19.5)	65.37 (21.3)	64.81 (22.0)	65.07 (21.7)	64.23 (22.9)	65.38 (23.1)	64.81 (23.0)	Gender: *F*(1) = 0.23, *p* = 0.63
Environment	81.66 (13.0)	80.54 (13.5)	81.08 (13.3)	76.33 (14.8)	73.28 (15.4)	74.75 (15.2)	76.08 (15.0)	74.29 (16.0)	75.17 (15.5)	Gender: *F*(1) = 28.73, *p* < 0.001

#### Gender

Pre-pandemic scores for women were between 77 and 81 and for men, the scores were 77–82. During the pandemic women scored between 65 and 73 in April and 65 and 74 in Dec/Jan. Men scored 65–76 and 64–77 in April and Dec/Jan, respectively. Detailed descriptive results for each gender are shown in Table 1. There was no interaction between gender and time period on physical QoL [*F*(2) = 0.7, *p* = 0.5]. Across all time points, women have poorer physical QoL than men [*F(*1) = 50.56, *p* < 0.001] and both men and women showed the same trend over time (i.e., pre-pandemic was better than pandemic and Dec/Jan was better than April).

There was no significant interaction between gender and time period on social QoL [*F*(2) = 0.65, *p* = 0.52], and there was no main effect of gender [*F*(1) = 0.23, *p* = 0.63], suggesting that both genders had similar social QoL at each time point.

There was no significant interaction between gender and time period on environmental QoL [*F*(2) = 2.91, = 0.55]. There was a significant main effect of gender [*F*(1) = 28.73, *p* < 0.001], with men reporting higher environmental QoL than women at all time points. The values are presented in Table 1.

#### Age

**Figure 1:**
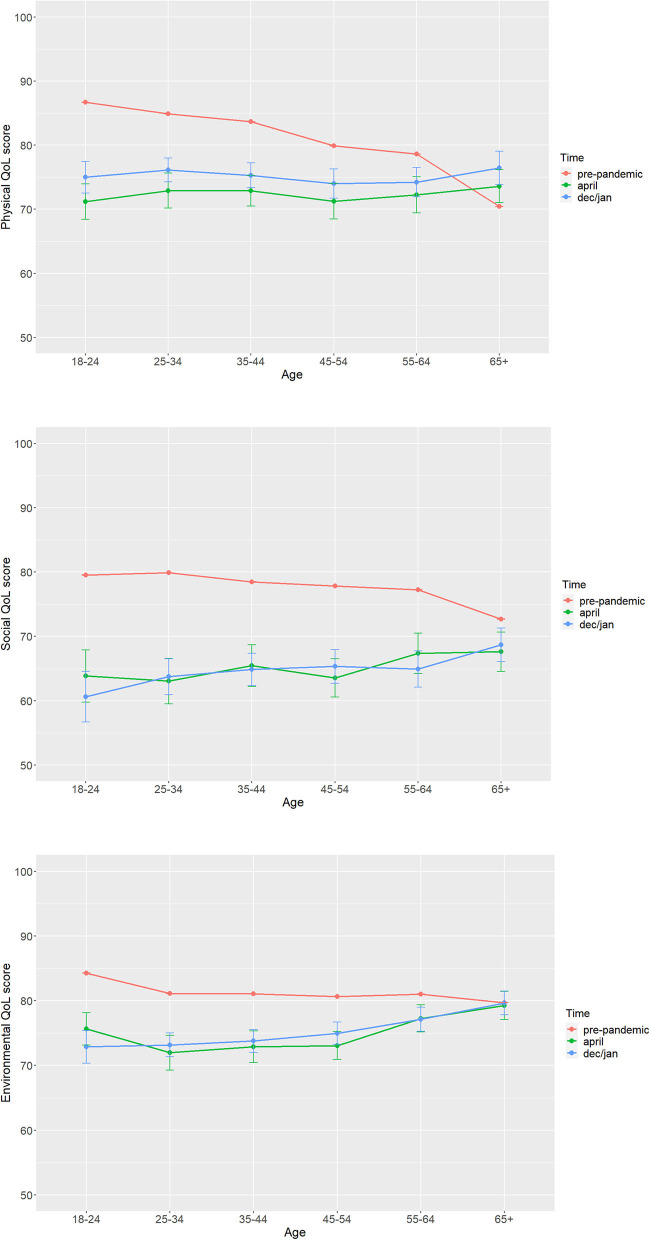
Mean quality of life scores for each age group at all three time points.

The results of quality of life in different age groups are shown in Figure 1. Generally, to use the youngest and oldest age groups as examples; the scores in 18–24-year-olds ranged from 79 to 86 pre-pandemic and 60 to 75 within the pandemic (including both time points), the scores in the over 65s ranged from 70 to 79 pre-pandemic and 67 to 79 within the pandemic. There were significant interactions between time point and age category on physical [*F*(10) = 19.55, *p* < 0.001], social [*F*(10) = 9.89, *p* < 0.001], and environmental [*F*(10) = 12.45, *p* < 0.001] quality of life.

Overall, all age groups except the over 65s show a decline from pre-pandemic to the pandemic in *physical* and *environmental* QoL, but no further decline within the pandemic (April vs. Dec/Jan). The over 65s actually show an improvement from pre-pandemic to Dec/Jan in *physical* QoL and no change across time in *environmental* QoL.

Regarding social QoL, all age groups show a decline from pre-pandemic to the pandemic, but no further decline within the pandemic. Significant *post-hoc* results are detailed in the Supplementary Table S3. As shown in Figure 1, the QoL decline from pre-pandemic to pandemic was larger in younger age groups.

### Research question 2

#### Urbanization

**Figure 2:**
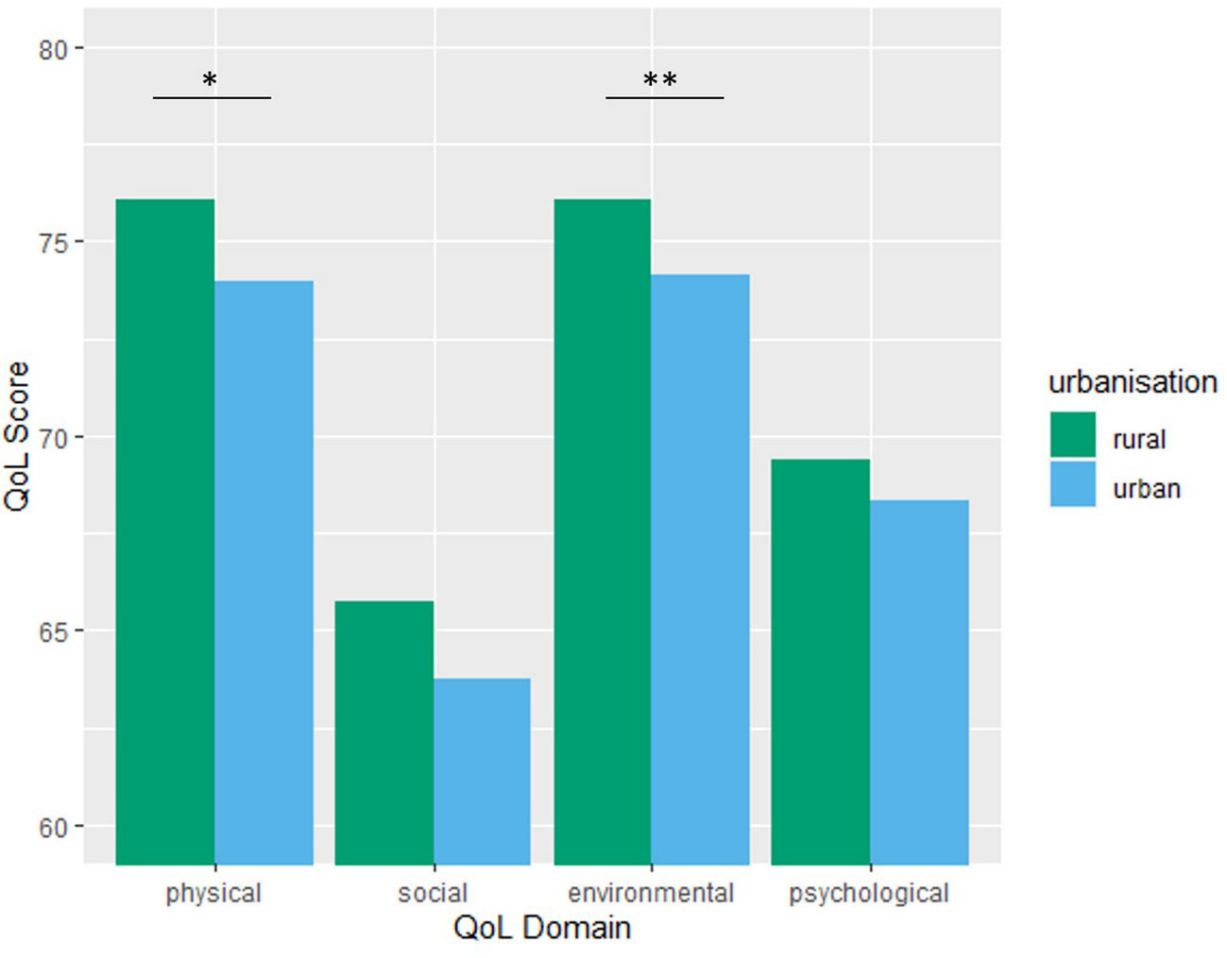
Mean quality of life scores according to urbanization level in the Dec/Jan lockdown sample. *p < 0.05, **p < 0.01. Urban >25000 inhabitants.

Pre-pandemic scores were between 78 and 81 in most rural locations and between 74 and 79 in most urban areas. During the pandemic, the QoL scores ranged from 65 to 76 in rural areas and 63 to 74 in urban settings. Figure 2 shows the QoL values for each urbanization category. In the Dec/Jan sample, there was a significant effect of living location on physical [*t*(1,503) = 2.23, *p* = 0.03] and environmental [*t*(1,503) = 2.43, *p* = 0.01] quality of life, with those living in rural areas showing higher scores than those living in cities (Figure 2). There was no effect of urbanization level on psychological [*t*(1,503) = 1.09, *p* = 0.28] nor social [*t*(1,503) = 1.7, *p* = 0.09] QoL.

In the pre-pandemic data, urbanization was coded differently (Statistik Austria, 2020) and was therefore not comparable with the Dec/Jan sample. However, the mean scores are presented in Table 2 for reference. In the pre-pandemic, sample low density was considered as fewer than 5,000 people, middle as 5,000–50,000 people, and high as more than 50,000.

**Table 2:** Mean (SD) quality of life scores in the pre-pandemic data according to population density (Moons et al., 2006).

	**Low**	**Middle**	**High**
Physical	80.2 (18.6)	80.1 (18.5)	78.9 (18.5)
Social	78.45 (19.1)	78.38 (19.1)	74.52 (20.1)
Environment	81.94 (12.5)	81.99 (13.1)	79.06 (14.1)
Psychological	80.51 (14.3)	80.4 (15.1)	78.07 (15.8)

## Discussion

The results show a decline in quality of life in both April 2020 and December 2020/January 2021, as compared with pre-pandemic data, in the Austrian general population. A further decline did not emerge within the pandemic, suggesting that the deterioration witnessed early in the COVID-19 pandemic lasted throughout the year, but did not worsen.

As expected, given that women typically exhibit poorer mental health than men (Riecher-Rössler, 2017), quality of life was generally lower in women than men. This was the case across both pre-pandemic and lockdown time points. The pandemic results corroborate studies on other aspects of mental health during the pandemic (Moghanibashi-Mansourieh, 2020; Wang et al., 2020), including psychological quality of life in the same sample (Pieh et al., 2020; Dale et al., 2021) and some studies do suggest that the mental health gender gap has further increased during the pandemic (Almeida et al., 2020; Dale et al., 2021), which emphasizes the need to take gender into account during mental health policy and decision-making (Smith, 2019). Despite the typical trend for poorer QoL in women, interestingly, men and women did not differ in the social quality of life at any time point. A study on the Brazilian population (Cruz et al., 2011) also found significantly lower WHOQOL-BREF scores in women than men in all domains except social, and in fact, Skevington et al. (2004) found women to have significantly higher social QoL scores than men in a 23-country study [but as shown in Purba et al. (2018)]. These results suggest that women prioritize this aspect of their lives more, regardless of the external conditions, highlighting the importance of social connections as a potential buffer to societal and psychological challenges (Santini et al., 2020).

Age also had an effect on the quality of life reported by the respondents. With the exception of the over 65s, all age groups show a decline from pre-pandemic to pandemic QoL. What is also clear is that the younger age groups have experienced a more pronounced drop in life quality than the older groups (Figure 1). This is in contrast to research prior to the pandemic, which shows that outside of lockdown periods, the quality of life typically declines with age (Skevington et al., 2004; Purba et al., 2018) and therefore, the pandemic is having an unprecedented effect on the lives of young people. This is supported by findings from Israel (Horesh et al., 2020), where the youngest age group (21–35 years) reported lower QoL than older age groups (>61 years). Combined with previous findings (Pieh et al., 2020, 2021b; Salari et al., 2020; Dale et al., 2021), these results indicate that the overall wellbeing of young people has been significantly affected, with the potential for long-term consequences. Concerns about the future (Salari et al., 2020) and missed opportunities are among the most relevant explanations for the higher burden on young people. Furthermore, older adults have been shown to exhibit greater emotional self-regulation and self-control (Flesia et al., 2020), which may have been protective factors for the mental health challenges during this time. Naturally the lockdown measures are essential to reduce the spread of a life-threatening virus, but given the multi-faceted nature of QoL, improvements in just one or two areas of life could lead to significant improvements in overall life quality, particularly for young people.

Finally, we also found some effects of the level of urbanization on the quality of life during the Dec/Jan lockdown. To our knowledge, this is the first study to investigate urbanization and quality of life during the COVID-19 pandemic. Those in villages showed higher physical and environmental QoL than those in large cities. In a large-scale study from 2009, Shucksmith et al. (2009) measured urban-rural differences in quality of life across the European Union and, while they used a different measure of QoL to the current study, found there to be very few urban-rural differences, particularly in subjective wellbeing, which is the most relevant measure for the current findings. Similarly, a study from Indonesia found no effect of urbanization on WHOQOL scores (Purba et al., 2018). However, our results do support the few other studies investigating mental wellbeing according to urbanization during the pandemic. In China, depression prevalence was higher in urban than rural participants (Gao et al., 2020), and in Turkey, depression scores were higher in urban areas and high urbanization was a risk factor for anxiety (Özdin and Bayrak Özdin, 2020). In contrast, a study in Cameroon found no association between urbanization, gender, or age on depression and fear during the pandemic but in this sample education level may have been more important (Siewe Fodjo et al., 2021). In summary, our results are congruent to Gao et al. (2020) and Özdin and Bayrak Özdin (2020), which were also conducted during the pandemic, but contrast with pre-pandemic urbanization studies (Shucksmith et al., 2009; Purba et al., 2018). Potential reasons for the divergences may be the COVID-19 situation, but different survey methods or cultural differences cannot be ruled out. Overall, these results suggest those in large cities are experiencing the physical and environmental restrictions of the lockdown to a greater extent than those in rural areas, despite Austrian cities, such as Vienna offering more green space than any other European city (Fair, 2020). Indeed, it has been well documented that green spaces are good for our mental health (White et al., 2021) and this may also be an explanation for the better QoL scores seen in rural areas in this study. In fact, a positive consequence of the pandemic may be the renewed interest in putting health and wellbeing at the forefront of urban planning (Mouratidis and Yiannakou, 2022) and our data support a need for this to occur. Further studies on cities with differing pandemic-restriction-compatible services, outdoor spaces, and opportunities for residents would elucidate these findings. Access possibilities to space and services should be promoted to alleviate negative QoL effects on city dwellers.

When interpreting the results, the following limitations have to be considered. It should be noted that the Dec/Jan sample had no subjects over 85, compared with the over 85s forming 12.42% of over 65s in the pre-pandemic sample. The lack of representation in the oldest of the population in the COVID-19 sample may have driven some of the effects seen in this sub-group, particularly in physical quality of life, which was observed to be better in the Dec/Jan sample than pre-pandemic and likely declines as mobility becomes reduced. Additionally, the urbanization results were not directly comparable with pre-pandemic data and therefore causal conclusions regarding the effects of the pandemic cannot be drawn. A further shortcoming is that no repeated measures from the same individuals were available, as new samples were drawn for each of the three surveys.

## Conclusion

Quality of life is a subjective evaluation of one's situation (WHO, 1996). Therefore, the poor quality of life scores here reflect how people see their lives in relation to their own goals, expectations, and standards during these unprecedented times. The pandemic has affected numerous areas of our daily lives and therefore mental health support should seek strategies to improve all domains of quality of life so as to improve the overall, multi-faceted natures of mental state, life satisfaction, and wellbeing. On a smaller scale, this should be especially directed to vulnerable sub-groups of the population, such as women and young people but on a larger scale, urban planning and policy should also take the quality of life into account as preparedness for future societal crises.

## Data availability statement

The raw data supporting the conclusions of this article will be made available by the authors, without undue reservation.

## Ethics statement

The studies involving human participants were reviewed and approved by Ethics Committee of the Danube University Krems, Austria. The patients/participants provided their written informed consent to participate in this study.

## Author contributions

Conceptualization and methodology: RD, SB, TP, and CP. Formal analysis, writing—original draft preparation, and visualization: RD. Investigation and data curation: RD and SB. Resources: CP. Writing—review and editing: RD, EH, TP, and CP. Supervision: TP and CP. Project administration: SB and EH. All authors contributed to the article and approved the submitted version.

## Conflict of interest

The authors declare that the research was conducted in the absence of any commercial or financial relationships that could be construed as a potential conflict of interest.

## Publisher's note

All claims expressed in this article are solely those of the authors and do not necessarily represent those of their affiliated organizations, or those of the publisher, the editors and the reviewers. Any product that may be evaluated in this article, or claim that may be made by its manufacturer, is not guaranteed or endorsed by the publisher.
