# Supplementary material for: Quality of life during the COVID-19 pandemic in Austria
Source: Front Psychol. 2022 Aug 1;13:934253. doi: 10.3389/fpsyg.2022.934253 (PMC9376461; doi:10.3389/fpsyg.2022.934253)
Supplement: Supplementary file 1 [file Data_Sheet_1.docx]

Supplementary Material

Table S1: Demographic characteristics of the COVID-19 lockdown samples.

|  | April sample | | Dec/Jan sample | |
| --- | --- | --- | --- | --- |
| Variable | N | % | N | % |
| Total | 1005 |  | 1505 |  |
| Gender |  |  |  |  |
| Women | 530 | 52.7 | 764 | 49.2 |
| Men  Other | 475  0 | 47.3  0 | 741  0 | 50.8  0 |
| Age |  |  |  |  |
| 18-24 | 118 | 11.7 | 153 | 10.2 |
| 25-34 | 166 | 16.5 | 279 | 18.5 |
| 35-44 | 185 | 18.4 | 289 | 19.2 |
| 45-54 | 222 | 22.1 | 326 | 21.7 |
| 55-64 | 181 | 18.0 | 272 | 18.1 |
| 65+ | 133 | 13.2 | 186 | 12.4 |
| Region |  |  |  |  |
| Burgenland | 35 | 3.5 | 58 | 3.9 |
| Lower Austria | 187 | 18.6 | 305 | 20.3 |
| Vienna | 218 | 21.7 | 346 | 23.0 |
| Carinthia | 66 | 6.6 | 103 | 6.8 |
| Styria | 149 | 14.8 | 221 | 14.7 |
| Upper Austria | 172 | 17.1 | 227 | 15.1 |
| Salzburg | 63 | 6.3 | 79 | 5.3 |
| Tyrol | 77 | 7.7 | 114 | 7.6 |
| Vorarlberg | 38 | 3.8 | 52 | 3.5 |
| Education |  |  |  |  |
| Low | 27 | 2.7 | 40 | 2.66 |
| Middle | 609 | 60.6 | 809 | 53.75 |
| High | 369 | 36.7 | 656 | 43.59 |

Table S2: Missing quotas from the Dec/Jan sample.

|  | Final % | Representative % | % Difference |
| --- | --- | --- | --- |
| Male 18-24 | 4.19 | 5.5 | 1.31 |
| Male 25-34 | 8.64 | 9.13 | 0.49 |
| Female 45-54 | 10.63 | 11.06 | 0.43 |
| Female 65+ | 5.58 | 6.56 | 0.98 |
| Vienna | 22.99 | 23.44 | 0.45 |
| Upper Austria | 15.08 | 16.69 | 1.6 |
| Salzburg | 5.25 | 6.31 | 1.06 |
| Tyrol | 7.57 | 8.5 | 0.93 |
| Vorarlberg | 3.46 | 4.44 | 0.98 |
| High Education | 43.59 | 53.06 | 9.47 |

Table S3: Significant results of Bonferonni corrected post-hoc tests for age.

| Physical |
| --- |
| 18 – 24: Pre-pandemic vs. April p<0.001, pre-pandemic vs. Dec/Jan p<0.001 |
| 25 – 34: Pre-pandemic vs. April p<0.001, pre-pandemic vs. Dec/Jan p<0.001 |
| 35 – 44: Pre-pandemic vs. April p<0.001, pre-pandemic vs. Dec/Jan p<0.001 |
| 45 – 54: Pre-pandemic vs. April p<0.001, pre-pandemic vs. Dec/Jan p<0.001 |
| 55 – 64: Pre-pandemic vs. April p<0.001, pre-pandemic vs. Dec/Jan p<0.001 |
| 65+: Pre-pandemic vs. Dec/Jan p=0.001 |
| Social |
| 18 – 24: Pre-pandemic vs. April p<0.001, pre-pandemic vs. Dec/Jan p<0.001 |
| 25 – 34: Pre-pandemic vs. April p<0.001, pre-pandemic vs. Dec/Jan p<0.001 |
| 35 – 44: Pre-pandemic vs. April p<0.001, pre-pandemic vs. Dec/Jan p<0.001 |
| 45 – 54: Pre-pandemic vs. April p<0.001, pre-pandemic vs. Dec/Jan p<0.001 |
| 55 – 64: Pre-pandemic vs. April p<0.001, pre-pandemic vs. Dec/Jan p<0.001 |
| 65+: Pre-pandemic vs. April p=0.002, pre-pandemic vs. Dec/Jan p=0.004 |
| Environmental |
| 18 – 24: Pre-pandemic vs. April p<0.001, pre-pandemic vs. Dec/Jan p<0.001 |
| 25 – 34: Pre-pandemic vs. April p<0.001, pre-pandemic vs. Dec/Jan p<0.001 |
| 35 – 44: Pre-pandemic vs. April p<0.001, pre-pandemic vs. Dec/Jan p<0.001 |
| 45 – 54: Pre-pandemic vs. April p<0.001, pre-pandemic vs. Dec/Jan p<0.001 |
| 55 – 64: Pre-pandemic vs. April p=0.001, pre-pandemic vs. Dec/Jan p<0.001 |
| 65+: NS |
